# Supplementary material for: Heat adaptation of phage T7 under an extended genetic code
Source: Virus Evol. 2021 Dec 1;7(2):veab100. doi: 10.1093/ve/veab100 (PMC8923235; doi:10.1093/ve/veab100)
Supplement: veab100_Supp [file veab100_supp.zip › Sequencing_Analysis.zip!/Sequencing_Analysis/S1/evidence/MC_SIDE_1_37.html]

BRESEQ :: Evidence


| Predicted mutation | | | | | | |
| --- | --- | --- | --- | --- | --- | --- |
| evidence | seq id | position | mutation | annotation | gene | description |
| MC JC | NC\_001604.1 | 1,253 | Δ1,489 bp | intergenic (–/–) | – / – | –/– |

| Missing coverage evidence... | | | | | | | | | | |
| --- | --- | --- | --- | --- | --- | --- | --- | --- | --- | --- |
|  |  |  | seq id | start | end | size | ←reads | reads→ | gene | description |
| \* | \* | ÷ | NC\_001604.1 | 1253 | 2741 | 1489 | 4499 [53] | [87] 4530 | *–/–* | –/– |

| New junction evidence | | | | | | | | | | | |
| --- | --- | --- | --- | --- | --- | --- | --- | --- | --- | --- | --- |
|  | | seq id | position | reads (cov) | reads (cov) | score | skew | freq | annotation | gene | product |
| \* | ? | NC\_001604.1 | = 1252 | 53 (0.010) | 4384 (1.080) | 395/580 | 0.0 | 98.5% | intergenic (–/–) | *–/–* | –/– |
|  | ? | NC\_001604.1 | 2742 = | 84 (0.020) |  |  |  |  | intergenic (–/–) | *–/–* | –/– |

Only 100 of 4499 total aligned reads displayed.  

`AACGTTTTCGACCACGCTTACGAAATGCTGAAAGAAAACATCCGTTATGATGACATCCGTGACACTGATGACCTGCACGATGCTATTCACATGGCTGCCGATAATGCAGTTCCGCACTACTACGCTGACATCTTTAGCGTAATGGCAAGTGAGGGCATTGACCTTGAGTTCGAAGACTCTGGTCTGATGCCTGACACCAAGGACGTAATCCGCATCCTGCAAGCGCGTATCTATGAGCAATTAACGATTGACCTCTGGGAAGACGCAGAAGACTTGCTCAATGAATACTTG‑‑‑‑‑‑‑‑‑GAGGAAGT‑‑‑‑‑‑‑‑‑CGAGGAGTACGAGGAGGATGAAGAGTAATGTCTACTACCAACGTGCAATACGGTCTGACCGCTCAAACTGTACTTTTCTATAGCGACATGGTGCGCTGTGGCTTTAACTGGTCACTCGCAATGGCACAGCTCAAAGAACTGTACGAAAACAACAAGGCAATAGCTTTAGAATCTGCTGAGTGATAGACTCAAGGTCGCTCCTAGCGAGTGGCCTTTATGATTATCACTTTACTTATGAGGGAGTAATGTATATGCTTACTATCGGTCTACTCACCGCTCTAGGTCTAG  >  NC_001604.1/952‑1538`  

                                                                                                                                                                                                                                                                                                                              `|`                                                                                                                                                                                                                                                                                                
            `agcgcTTACGAAATGCTGAAAGAAAACATCCGTTATGATGACATCCGTGACACTGATGACCTGCACGATGCTATTCACATGGCTGCCGATAATGCAGTTCCGCACTACTACGCTGACATCTTTAGCGTAATGGCAAGTGAGGGCATTGACCTTGAGTTCGAAGACTCTGGTCTGATGCCTGACACCAAGGACGTAATCCGCATCCTGCAAGCGCGTATCTATGAGCAATTAACGATTGACCTCTGGGAAGACGCAGAAGACTTGCTCAATGAATACTTG‑‑‑‑‑‑‑‑‑GAGGAAGT‑‑‑‑‑‑‑‑‑CGcacgacagaaag                                                                                                                                                                                                                                                                                    <  1:264981‑M1/299‑13 (MQ=255)`  
              `cGCTTACGAAATGCTGAAAGAAAACATCCGTTATGATGACATCCGTGACACTGATGACCTGCACGATGCTATTCACATGGCTGCCGATAATGCAGTTCCGCACTACTACGCTGACATCTTTAGCGTAATGGCAAGTGAGGGCATTGACCTTGAGTTCGAAGACTCTGGTCTGATGCCTGACACCAAGGACGTAATCCGCATCCTGCAAGCGCGTATCTATGAGCAATTAACGATTGACCTCTGGGAAGACGCAGAAGACTTGCTCAATGAATACTTG‑‑‑‑‑‑‑‑‑GAGGAAGT‑‑‑‑‑‑‑‑‑CGcacgacctgtctct                                                                                                                                                                                                                                                                                  >  1:210371‑M1/1‑287 (MQ=255)`  
                             `acaggaaaACATCCGTTATGATGACATCCGTGACAATGATGACCTGCACGATGCTATTCACATGGCTGCCGATAATGCAGTTCCGCACTACTACGCTGACATCTTTAGCGTAATGGCAAGTGAGGGCATTGACCTTGAGTTCGAAGACTCTGGTCTGATGCCTGACACCAAGGACGTAATCCGCATCCTGCAAGCGCGTATCTATGAGCAATTAACGATTGACCTCTGGGAAGACGCAGAAGACTTGCTCAATGAATACTTG‑‑‑‑‑‑‑‑‑GAGGAAGT‑‑‑‑‑‑‑‑‑Cgaggaaatcgcacgacagaaagaaattgac                                                                                                                                                                                                                                                                   <  1:354899/297‑26 (MQ=255)`  
                             `aaaagaaaACATCCGTTATGATGACATCCGGGACACTGATGACCTGCACGAGGCTATTCACATGGCTGCCGATAATGCAGTTCCGCACTACTACGCTGACATCTTTAGCGTAATGGCAAGTGAGGGCATTGACCTTGAGTTCGAAGACTCTGGTCTGATGCCTGACACCAAGGACGTAATCCGCATCCTGCAAGCGCGTATCTATGAGCAATTAACGATTGACCTCTGGGAAGACGCAGAAGACTTGCTCAATGAATACTTG‑‑‑‑‑‑‑‑‑GAGGAAGT‑‑‑‑‑‑‑‑‑Cgaggaagtcgcacgacagaaagaaattgac                                                                                                                                                                                                                                                                   <  1:654200/300‑26 (MQ=255)`  
                               `aagaaaACATCCGTTATGATGACATCCGTGACACTGATGACGTGCACGATGCTATTCACATGGCTGCCGATAATGCAGTTCCACACTACTACGCTGACATCTTTAGGGTAATGGCAAGTGAGGGCATTGACCTTGAGTTCGAAGACTCTGGTCTGATGCCTGACACCAAGGACGTAATCCGCATCCTGCAAGCGCGTATCTATGAGCAATTAACGATTGACCTCTGGGAAGACGCAGAAGACTTGCTCAATGAATACTTG‑‑‑‑‑‑‑‑‑GAGGAAGT‑‑‑‑‑‑‑‑‑CGcacgacagaaagaaattgaccgcgctaaggc                                                                                                                                                                                                                                                                 <  1:622913‑M1/301‑32 (MQ=255)`  
                               `aagaaaACATCCGTGATGATGACATCCGTGACACTGATGACCTGCACGATGCTATTAACATGGATGCCGATAATGCAGTTTCGCACTACTACGCTGACATCTTTAGCGTAATGGAAAGTGAGGGCATTGACCTCGAGTTCGAAGACTCTGGTCTGATGCCTGACACCAAGGACGTAATCCGCATCCTGCAAGCGCGTATCTATGAGCAATTAACGATTGACCTCTGGGAAGACGCAGAAGACTTGCTCAATGAATACTTG‑‑‑‑‑‑‑‑‑GAGGAAGT‑‑‑‑‑‑‑‑‑CGcacgacagaaagaaattgaccgcgataaggc                                                                                                                                                                                                                                                                 <  1:42971‑M1/301‑32 (MQ=255)`  
                                `agaaaACATCCGTTATGATGACATCCGGTACACTGATGACCTGCACGATGCTATTCACATGGCTGCCGATAATGCAGTTCCGCACTACTACGCTGACATCGTTAGCGTAATGGCAAGTGAGGGCATTGACCTTGAGTTCGAAGACTCTGGTCTGATGCCTGACACCAAGGACGTAATCCGCATCCTGCAAGCGCGTATCTATGAGCAATTAACGATTGACCTCTGGGAAGACGCAGAAGACTTGCTCAATGAATACTTG‑‑‑‑‑‑‑‑‑GAGGAAGT‑‑‑‑‑‑‑‑‑CGcacgacagaaagaaattgaccgcgctaaggcc                                                                                                                                                                                                                                                                <  1:61685‑M1/301‑33 (MQ=255)`  
                                           `gTTATGATGACATCCGTGACACTGATGACCTGCACGATGCTATTCACATGGCTGCCGATAATGCAGTTCCGCACTACTACGCTGACATCTTTAGCGTAATGGCAAGTGAGGGCATTGACCTTGAGTTCGAAGACTCTGGTCTGATGCCTGACACCAAGGACGTAATCCGCATCCTGCAAGCGCGTATCTATGAGCAATTAACGATTGACCTCTGGGAAGACGCAGAAGACTTGCTCAATGAATACTTG‑‑‑‑‑‑‑‑‑GAGGAAGT‑‑‑‑‑‑‑‑‑CGcacgacagaaagaacttgaccgcgctaaggccggtaaagaacg                                                                                                                                                                                                                                                     >  1:543974‑M1/1‑258 (MQ=255)`  
                                            `ttATGATGACATCCGTGACACTGATGACCTGCACGATGCTATTCACATGGCTGCCGATAATGCAGTTCCGCACTACTACGCTGACATCTTTAGCGTAATGGCAAGTGAGGGCATTGACCTTGAGTTCGAAGACTCTGGTCTGATGCCTGACACCAAGGACGTAATCCGCATCCTGCAAGCGCGTATCTATGAGCAATTAACGATTGACCTCTGGGAAGACGCAGAAGACTTGCTCAATGAATACTTG‑‑‑‑‑‑‑‑‑GAGGAAGT‑‑‑‑‑‑‑‑‑CGcacgacagaaagaaattgaccgcgctaaggcccgtaaagaacgt                                                                                                                                                                                                                                                    >  1:379912‑M1/1‑257 (MQ=255)`  
                                               `cagtgacatCCTTGACACTGATGACCTGCACGATGCTATTCCCATGGCTGCCGATAATGCAGTTCCGCACTACTACGCTGACATCTTTAGCGTAATGGCAAGTGAGGGCATTGACCTTGAGTTCGAAGACTCTGGTCTGATGCCTGACACCAAGGACGTAATCCGCATCCTGCAAGCGCGTATCTATGAGCAATTAACGATTGACCTCTGGGAAGACGCAGAAGACTTGCTCAATGAATACTTG‑‑‑‑‑‑‑‑‑GAGGAAGT‑‑‑‑‑‑‑‑‑CGcacgacagaaagaaattgaccgcgctaaggcccgtaaagaacgtcac                                                                                                                                                                                                                                                 <  1:18731‑M1/298‑48 (MQ=255)`  
                                                `gatgaCATCCGTGACACTGATGACCTGCACGATGCTATTCACATGGCTGCCGATAATGCAGTTCCGCACTACTACGCTGACATCTTTAGCGTAATGGCAAGTGAGGGCATTGACCTTGAGTTCGAAGACTCTGGTCTGATGCCTGACACCAAGGACGTAATCCGCATCCTGCAAGCGCGTATCTATGAGCAATTAACGATTGACCTCTGGGAAGACGCAGAAGACTTGCTCAATGAATACTTG‑‑‑‑‑‑‑‑‑GAGGAAGT‑‑‑‑‑‑‑‑‑CGcacgacagaaagaaattgaccgcgctaaggcccgtaaagaacgtcacg                                                                                                                                                                                                                                                <  1:571025‑M1/301‑49 (MQ=255)`  
                                                 `atgaCATCCGTGACACTGATGACCTGAACGATGCTATTCACATGGCTGCCGATAATGCAGTTCCGCACTACTACGCTGACATCTTTAGCGAAATGGCAAGTGAGGGCATTGACCTTGAGTTCGAAGACTCTGGTCTGATGCCTGACAACAAGGACGTAATCCGCATCCTGCAAGCGCGTATCTATGAGCAATTAACGATTGACCTCTGGGAAGACGCAGAAGACTTGCTCAATGAATACTTG‑‑‑‑‑‑‑‑‑GAGGAAGT‑‑‑‑‑‑‑‑‑CGcacgacagaaagaaattgaccgcgctaaggcccgtaaagaacgtcacgg                                                                                                                                                                                                                                               >  1:306017‑M1/1‑252 (MQ=255)`  
                                                      `aTCCGTGACACTGATGACCTACACGATGCTATTCACATGGCTGCCGATAATGCAGTTCCGCACTACTACGCTGACATCCTTAGCGTAATGGCAAGTGAGGGCATTGACCTTGAGTTCGAAGACTCTGGTCTGATGCCTGACACCAAGGACGTAATCCGCATCCTGCAAGCGCGTATCTATGAGCAATTAACGATTGACCTCTGGGAAGACGCAGAAGACTTGCTCAATGAATACTTG‑‑‑‑‑‑‑‑‑GAGGAAGT‑‑‑‑‑‑‑‑‑CGcacgacagaaagaaattgaccgcgctaaggcccgtaaagaacgtcacgaggggc                                                                                                                                                                                                                                          >  1:59025‑M1/1‑247 (MQ=255)`  
                                                             `acacTGATGACCTGCACGATGCTATTCACATGGCTGCCGATAATGCAGTTCCGCACTACTACGCTGACATCTTTAGCGTAATGGCAAGTGAGGGCATTGACTTTGAGTTCGAAGACTCTGGTCTGATGCCTGACACCAAGGACGTAATCCGCATCCTGCAAGCGCGTATCTATGAGCAATTAACGATTGACCTCTGGGAAGACGCAGAAGCCTTGCTCAATGAATACTTG‑‑‑‑‑‑‑‑‑GAGGAAGT‑‑‑‑‑‑‑‑‑CGcacgacagaaagaaattgaccgcgataaggcccgtaaagcacgtcacgaggggcgcttaga                                                                                                                                                                                                                                   >  1:100235‑M1/1‑240 (MQ=255)`  
                                                               `acTGATGACCTGCACGATGCTATTCACATGGCTGCCGATAATGCAGTTCCGCACTACTACGCTGACATCTTTAGCGTAATGGCAAGTGAGGGCATTGACCTTGAGTTCGAAGACTCTGGTCTGATGCCTGACACCAAGGACGTAATCCGCATCCTGCAAGCGCGTATCTATGAGCAATTAACGATTGACCTCTGGGAAGACGCAGAAGACTTGCTCAATGAATACTTG‑‑‑‑‑‑‑‑‑GAGGAAGT‑‑‑‑‑‑‑‑‑CGcacgacagaaagaaattgaccgcgctaaggcccgtaaagaacgtcacgaggggcgcttagagg                                                                                                                                                                                                                                 <  1:457322‑M1/301‑64 (MQ=255)`  
                                                               `acTGATGACCTGCACGATGCTATTCACATGGCTGCCGATAATGCAGTTCCGCACTACTACGCTGACATCTTTAGCGTAATGGCAAGTGAGGGCATTGACCTTGAGTTCGAAGACTCTGGTCTGATGCCTGACACCAAGGACGTAATCCGCATCCTGCAAGCGCGTATCTATGAGCAATTAACGATTGACCTCTGGGAAGACGCAGAAGACTTGCTCAATGAATACTTG‑‑‑‑‑‑‑‑‑GAGGAAGT‑‑‑‑‑‑‑‑‑CGcacgacagaaagaaattgaccgcgctaaggcccgtaaagaacgtcacgaggggcgcttagagg                                                                                                                                                                                                                                 <  1:147043‑M1/301‑64 (MQ=255)`  
                                                                `cTGATGACCTGCACGATGCTATTCACATGGCTGCCGATAATGCAGTTCCGCACTACTACGCTGACATCTTTAGCGTAATGGCAAGTGAGGGCATTGACCTTGAGTTCGAAGACTCTGGTCTGATGCCTGACACCAAGGACGTAATCCGCATCCTGCAAGCGCGTATCTATGAGCAATTAACGATTGACCTCTGGGAAGACGCAGAAGACTTGCTCAATGAATACTTG‑‑‑‑‑‑‑‑‑GAGGAAGT‑‑‑‑‑‑‑‑‑CGcacgacagaaagaaattgaccgcgctaaggcccgtaaagaacgtcacgaggggcgcttagaggc                                                                                                                                                                                                                                >  1:505537‑M1/1‑237 (MQ=255)`  
                                                                `cTGATGACCTGCACGATGCTATTCACATGGCTGCCGATAATGCAGTTCCGCACTACTACGCTGACATCTTTAGCGTAATGGCAAGTGAGGGCATTGACCTTGAGTTCGAAGACTCTGGTCTGATGCCTGACACCAAGGACGTAATCCGCATCCTGCAAGCGCGTATCTATGAGCAATTAACGATTGACCTCTGGGAAGACGCAGAAGACTTGCTCAATGAATACTTG‑‑‑‑‑‑‑‑‑GAGGAAGT‑‑‑‑‑‑‑‑‑CGcacgacagaaagaaattgaccgcgctaaggcccgtaaagaacgtcacgaggggcgcttagactg                                                                                                                                                                                                                                >  1:215643‑M1/1‑237 (MQ=255)`  
                                                                  `gatgaCCTGCACGATGCTATTCACATGGCTGCCGATAATGCAGTTCCGCACTACTACGCTGACATCTTTAGCGTAATGGCAAGTGAGGGCATTGACCTTGAGTTCGAAGACTCTGGTCTGATGCCTGACACCAAGGACGTAATCCGCATCCTGCAAGCGCGTATCTATGAGCAATCAACGATTGACCTCTGGGAAGACGCAGAAGACTTGCTCAATGAATACTTG‑‑‑‑‑‑‑‑‑GAGGAAGT‑‑‑‑‑‑‑‑‑CGcacgacagaaagaaattgaccgcgctaaggcccgtaaagaacgtcacgaggggcgcttagaggcct                                                                                                                                                                                                                              >  1:182962‑M1/1‑235 (MQ=255)`  
                                                                  `gatgaCCTGCACGATGCTATTAACATGGCTGCCGATAATGCAGTTCCGCACTACTACGCTGACATCTTTAGCGTAATGGCAAGTGAGGGCATTGACCTTGAGTTCGAAGACTCTGGTCTGATGCCTGACACCAAGGACGTAATCCGCATCCTGCAAGCGCGTATCTATGAGCAATTAACGATTGACCTCTGGGAAGACGCAGAAGACTTGCTCAATGAATACTTG‑‑‑‑‑‑‑‑‑GAGGAAGT‑‑‑‑‑‑‑‑‑CGcacgacagaaagaaattgaccgcgctaaggcccgtaaagaacgtcacgaggggcgcttagaggctg                                                                                                                                                                                                                              >  1:311794‑M1/1‑235 (MQ=255)`  
                                                                              `gATGCTATTCACATGGCGGCCGATAATGCAGTTCCGCACTACTACGCTGACATCTTTAGCGTAATGGCAAGTGAGGGCATTGACCTTGAGTTCGAAGACTCTGGTATGATGCCTGACACCAAGGACGTAATCCGCATCCTGCAAGCGAGTATCTATGAGCAATTAACGATTGACCTCTGGAAAGACGCAGAAGACTTGCTCAATGAATACTTG‑‑‑‑‑‑‑‑‑GAGGAAGT‑‑‑‑‑‑‑‑‑CGcacgacagaaagaaattgaccgcgctaaggcccgtaaagaacgtcacgaggggcgcttagaggcacgcagattcaaac                                                                                                                                                                                                                  <  1:138906‑M1/301‑79 (MQ=255)`  
                                                                              `gATGCTAGTCACATGGCGGCAGATAATGAAGGTACGCAATACGACGCTGACATCTGTAGCGTAACGGCAAGAGAGGGCATTGACCTGGTGTTCGAAGACTCTGGTCTGATGCCTGACACCAAGGACGTAATCCTCATCCTGCAAGCGCGTATCTATGAGCAATTAACGATTGACCGCTGGGAAGACGCAGAAGACTTGCTCAATGAATACTTG‑‑‑‑‑‑‑‑‑GAGGAAGT‑‑‑‑‑‑‑‑‑CGcacgacagaaagaaattgacctcgctaaggcccgtaaagaacgtcacgaggggcgcttagaggcacgcagattcaaac                                                                                                                                                                                                                  <  1:270672‑M1/301‑79 (MQ=255)`  
                                                                               `aTGCTATTCACATGGCTGCCGATAATGCAGTTCCGCACTACTACGCTGACATCTTTAGCGTAATGGCAAGTGAGGGCATTGACCTTGAGTTCGAAGACTCTGGTCTGATGCCTGACACCAAGGACGTAATCCGCATCCTGCAAGCGCGTATCTATGAGCAATTAACGATTGACCTCTGGGAAGACGCAGAAGACTTGCTCAATGAATACTTG‑‑‑‑‑‑‑‑‑GAGGAAGT‑‑‑‑‑‑‑‑‑CGcacgacagaaagaaattgaccgcgctaaggcccgtaaagaacgtcacgaggggcgcttagaggcacgcagattcaaacg                                                                                                                                                                                                                 >  1:52275‑M1/1‑222 (MQ=255)`  
                                                                               `aTGCTATTCACATGGCTGCCGATAATGCAGTTCCGCACTACTACGCTGACATCTTTAGCGTAATGGCAAGTGAGGGCATTGACCTTGAGTTCGAAGACTCTGGTCTGATGCCTGACACCAAGGACGTAATCCGCATCCTGCAAGCGCGTATCTATGAGCAATTAACGATTGACCTCTGGGAAGACGCAGAAGACTTGCTCAATGAATACTTG‑‑‑‑‑‑‑‑‑GAGGAAGT‑‑‑‑‑‑‑‑‑CGcacgacagaaagaaattgaccgcgctaaggcccgtaaagaacgtcacgaggggcgcttagaggaacgcagattcaaacg                                                                                                                                                                                                                 >  1:529282‑M1/1‑222 (MQ=255)`  
                                                                                    `aTTCACATGGCTGCCGATAATGCAGTTCCGCACTACTACGCTGACATCTTTAGCGTAATGGCAAGTGAGGGCATTGACCTTGAGTTCGAAGACTCTGGTCTGATGCCTGACACCAAGGACGTAATCCGCATCCTGCAAGCGCGTATCTATGAGCAATTAACGATTGACCTCTGGGAAGACGCAGAAGACTTGCTCAATGAATACTTG‑‑‑‑‑‑‑‑‑GAGGAAGT‑‑‑‑‑‑‑‑‑CGcacgacagaaagaaattgaccgcgctaaggcccgtaaagaacgtcacgaggggcgcttagaggcacgcagattcaaacgtcgca                                                                                                                                                                                                            >  1:322545‑M1/1‑217 (MQ=255)`  
                                                                                        `acaTGGCTGCCGATAATGCAGTTCCGCACTACTACGCTGACATCTTTAGCGTAATGGCAAGTGAGGGCATTGACATTGAGTTCGAAGACTCTGGTCTGATGCCTGACACCAAGGACGTAATCCGCATCGTGCAAGCGCGTATCTATGAGCAATTAACGATTGACCTCTGGGAAGACGCAGAAGACTTGCTCAATGAATACTTG‑‑‑‑‑‑‑‑‑GAGGAAGT‑‑‑‑‑‑‑‑‑CGcacgacagaaagaaattgaccgcgctaaggcccgtaaagaacgtcacgaggggcgcttagagtcacgcagattcaaacgtcgcaaccg                                                                                                                                                                                                        <  1:198826‑M1/301‑89 (MQ=255)`  
                                                                                             `gatgcCGATAATGCAGTTCCGCACTACTACGCTGACATCTTTAGCGTAATGGCAAGTGAGGGCATTGACCTTGAGTTCGAAGACTCTGGTCTGATGCCTGACACCAAGGACGTAATCCGCATCCTGCAAGCGCGTATCTATGAGCAATTAACGATTGACCTCTGGGAAGACGCAGAAGACTTGCTCAATGAATACTTG‑‑‑‑‑‑‑‑‑GAGGAAGT‑‑‑‑‑‑‑‑‑CGcacgacagaaagaaattgaccgcgctaaggcccgtaaagaacgtcacgaggggcgcttagaggcacgcagattcaaacgtcgcaaccgcaagg                                                                                                                                                                                                   >  1:493813‑M1/3‑208 (MQ=255)`  
                                                                                             `gCTGCCGATAATGAAGTTCCGCACTACTACGCTGACATCTTTAGCGTAATGGCAAGTGAGGGCATTGACCTTGAGTTCGAAGACTCTGGTCTGATGCCTGACACCAAGGACGTAATCCGCATCCTGCAAGCGCGTATCTAAGAGCAATTAACGATTGACCTCTGGGAAGACGCAGAAGACTTGCTCAATGAATACTTGGAGGAAGTCGAGGAAGT‑‑‑‑‑‑‑‑‑CGcacgacagaaagaaattgaccgcgctaaggcccgtaaagaacgtcacgaggggcgcttagaggcacgcagattcaaacgtcgca                                                                                                                                                                                                            >  1:602865‑M1/1‑217 (MQ=255)`  
                                                                                                    `aTAATGCAGTTCCGCACTACTACGCTGACATCTTTAGCGTAATGGCAAGTGAGGGCATTGACCTTGAGTTCGAAGACTCTGGTCTGATGCCTGACACCAAGGACGTAATCCGCATCCTGCAAGCGCGTATCTATGAGCAATTAACGATTGACCTCTGGGAAGACGCAGAATACTTGCTCAATGAATACTTG‑‑‑‑‑‑‑‑‑GAGGAAGT‑‑‑‑‑‑‑‑‑CGcacgacagaaagaaattgaccgcgctaaggcccgtaaagaacgtcacgaggggcgcttagaggcacgcagatttaaacgtcgccaccgcacggcacgtac                                                                                                                                                                                            >  1:393342‑M1/1‑201 (MQ=255)`  
                                                                                                       `aTGCAGTTCCGCACTACTACGCTGACATCTTTAGCGTAATGGCAAGTGAGGGCATTGACCTTGAGTTCGAAGACTCTGGTCTGATGCCTGACACCAAGGACGTAATCCGCATCCTGCAAGCGCGTATCTATGAGCAATTAACGATTGACCTCTGGGAAGACGCAGAAGACTTGCTCAATGAATACTTG‑‑‑‑‑‑‑‑‑GAGGAAGT‑‑‑‑‑‑‑‑‑CGcacgacagaaagaaattgaccgcgctaaggcccgtaaagaacgtcacgaggggcgcttagaggcacgcagattcaaacgtcgcaaccgcaaggcacgtaaagc                                                                                                                                                                                         >  1:194981‑M1/1‑198 (MQ=255)`  
                                                                                                       `aTGCAGTTCCGCACTACTACGCTGACATCTTTAGCGTAATGGCAAGTGAGGGCATTGACCTTGAGTTCGAAGACTCTGGTCTGATGCCTGACACCAAGGACGTAATCCGCATCCTGCAAGCGCGTATCTATGAGCAATTAACGATTGACCTCTGGGAAGACGCAGAAGACTTGCTCAATGAATACTTG‑‑‑‑‑‑‑‑‑GAGGAAGT‑‑‑‑‑‑‑‑‑CGcacgacagaaagaaattgaccgcgctaaggcccgtaaagaacgtcacgaggggcgcttagaggcacgcagattcaaacgtcgcaaccgcaaggcacgtaaagc                                                                                                                                                                                         >  1:176329‑M1/1‑198 (MQ=255)`  
                                                                                                          `cAGTTCCGCACTACTACGCTGACATCTTTAGCGTAATGGCAAGTGAGGGCATTGACCTTGAGTTCGAAGACTCTGGTCTGATGCCTGACACCAAGGACGTAATCCGCATCCTGCAAGCGCGTATCTATGAGCAATTAACGATTGACCTCTGGGAAGACGCAGAAGACTTGCTCAATGAATACTTGGAGGAAGTCGAGGAAGT‑‑‑‑‑‑‑‑‑CGcacgacagaaagaaattgaccgcgctaaggcccgtaaagaacgtcacgaggggcgcttagaggcacgcagattcaaacgtcgcaaccgcaaggcacg                                                                                                                                                                                               >  1:480583‑M1/1‑204 (MQ=255)`  
                                                                                                           `cgtTCCGCACTCCTACGCTGACATCTTTAGCGTAATGGCAAGTGAGGGCATTGACCTTGAGTTCGAAGACTCTGGTCTGATGCCTGACACCAAGGACGTAATCCGCATCCTGCAAGCGCGTATCTATGAGCAATTAACGATTGACCTCTGGGAAGACGCAGAAGACTTGCTCAATGAATACTTG‑‑‑‑‑‑‑‑‑GAGGAAGT‑‑‑‑‑‑‑‑‑CGcacgacagaaagaaattgaccgcgctaaggcccgtaaagaacgtcacgaggggcgcttagaggcacgcagattcaaacgtcgcaaccgcaaggcacgtaaagcacac                                                                                                                                                                                     <  1:240915‑M1/300‑108 (MQ=255)`  
                                                                                                            `gTTCCGCACTACTACGCTGACATCTTTAGCGTAATGGCAAGTGAGGGCATTGACCTTGAGTTCGAAGACTCTGGTCTGATGCCTGACACCAAGGACGTAATCCGCATCCTGCAAGCGCGTATCTATGAGCAATTAACGATTGACCTCTGGGAAGACGCAGAAGACTTCCTCAATGAATACTTG‑‑‑‑‑‑‑‑‑GAGGAAGT‑‑‑‑‑‑‑‑‑CGcacgacagaaagaaattgaccgcgctaaggcccgtaaagaacgtcacgaggggcgcttagaggcacgcagattcaaacgtcgcaaccgcaaggcacgtaaagcacaca                                                                                                                                                                                    >  1:407679‑M1/1‑193 (MQ=255)`  
                                                                                                                 `gCACTACTACGCTGACATCTTTAGCGTAATGGCAAGTGAGGGCATTGACCTTGAGTTCGAAGACTCTGGTCTGATGCCTGACACCAAGGACGTAATCCGCATCCTGCAAGCGCGTATCTATGAGCAATTAACGATTGACCTCTGGGAAGACGCAGAAGACTTGCTCAATGAATACTTG‑‑‑‑‑‑‑‑‑GAGGAAGT‑‑‑‑‑‑‑‑‑CGcacgacagaaagaaattgaccgcgctaaggcccgtaaagaacgtcacgaggggcgcttagaggcacgcagattcaaacgtcgcaaccgcaaggcacgtaaagcacacaaagct                                                                                                                                                                               >  1:132053‑M1/1‑188 (MQ=255)`  
                                                                                                                 `aatggccagcgctgacatcTTTAGCGTAATGGGAAGGGAGGACATTGACCTTGATGTCGAAGACTCTGGTCTGATGCCTGACACCAAGGACGTAATCCGCATCCTGCAAGCGCGTATCTATGAGCAATTAACGATTGAACTCTGGGAAGACGCAGAAGACTTGCTCAATGAATACTTG‑‑‑‑‑‑‑‑‑GAGGAAGT‑‑‑‑‑‑‑‑‑CGcacgacagaaagaaattgaccgcgctaaggcccttaaagaacgtcacgaggggcgcttagaggcacgcagattcaaacgtcgcaaccgaaaggcacgtaaagcacacaaagct                                                                                                                                                                               <  1:70150‑M1/292‑114 (MQ=255)`  
                                                                                                                    `ctactaCGCTGACATCTTTAGCGTAATGGCAAGTGAGGGCATTGACCTTGAGTTCGAAGACTCTGGTCTGATGCCTGACACCAAGGACGTAATCCGCATCCTGCAAGCGCGTATCTATGAGCAATTAACGATTGACCTCTGGGAAGACGCAGAAGACTTGCTCAATGAATACTTGGAGGAAGTCGAGGAAGT‑‑‑‑‑‑‑‑‑CGcacgacagaaagaaattgaccgcgctaaggcccgtaaagaacgtcacgaggggcgattagaggcacgaagattaaaacgtcgcaaccgcaaggaacgtaaagcacaa                                                                                                                                                                                     >  1:47481‑M1/1‑194 (MQ=255)`  
                                                                                                                    `ctactaCGCTGACATCTTTAGCGTAATGGCAAGTGAGGGCATTGACCTTGAGTTCGAAGACTCTGGTCTGATGCCTGACACCAAGGACGTAATCCGCATCCTGCAAGCGCGTATCTATGAGCAATTAACGATTGACCTCTGGGAAGACGCAGAAGACTTGCTCAATGAATACTTG‑‑‑‑‑‑‑‑‑GAGGAAGT‑‑‑‑‑‑‑‑‑CGcacgacagaaagaaattgaccgcgctaaggcccgtaaagaacgtcacgaggggcgcttagaggcacgcagattcaaacgtcgcaaccgcaaggcacgtaaagcacacaacgctctg                                                                                                                                                                            >  1:418667‑M1/1‑185 (MQ=255)`  
                                                                                                                            `cTGACATCTTTAGCGTAATGGCAAGTGAGGGCATTGACCTTGAGTTCGAAGACTCTGGTCTGATGCCTGACACCAAGGACGTAATCCGCATCCTGCAAGCGCGTATCTATGAGCAATTAACGATTGACCTCTGGGAAGACGCAGAAGACTTGCTCAATGAATACTTG‑‑‑‑‑‑‑‑‑GAGGAAGT‑‑‑‑‑‑‑‑‑CGcacgacagaaagaaattgaccgcgctaaggcccgtaaagaacgtcacgaggggcgcttagaggcacgcagattcaaacgtcgcaaccgcaaggcacgtaaagcacacaaagctaagcgcgaaag                                                                                                                                                                    <  1:10727‑M1/301‑125 (MQ=255)`  
                                                                                                                              `gACATCTTTAGCGTAATGGCAAGTGAGGGCATTGACCTTGAGTTCGAAGACTCTGGTCTGATGCCTGACACCAAGGACGTAATCCGCATCCTGCAAGCGCGTATCTATGAGCAATTAACGATTGACCTCTGGGAAGACGCAGAAGACTTGCTCAATGAATACTTGGAGGAAGTCGAGGAAGT‑‑‑‑‑‑‑‑‑CGcacgacagaaagaaattgaccgcgctaaggcccgtaaagaacgtcacgaggggcgcttataggcacgcagattcaaacgtagcaacagcaatgaacgtaaagaacacaaagctaagg                                                                                                                                                                           >  1:349415‑M1/1‑184 (MQ=255)`  
                                                                                                                              `gACATCTTTAGCGTAATGGCAAGTGAGGGCATTGACCTTGAGTTCGAAGACTCTGGTCTGATGCCTGACACCAAGGACGTAATCCGCATCCTGCAAGCGCGTATCTATGAGCAATTAACGATTGACCTCTGGGAAGACGCAGAAGACTTGCTCAATGAATACTTG‑‑‑‑‑‑‑‑‑GAGGAAGT‑‑‑‑‑‑‑‑‑CGcacgacagaaagaaattgaccgcgctaaggcccgtaaagaacgtcacgaggggcgcttagaggcacgcagattcaaacgtcgcaaccgcaaggcacgtaaagcacacaaagctaagctcgaaagaa                                                                                                                                                                  >  1:45180‑M1/1‑175 (MQ=255)`  
                                                                                                                                 `aTCTTTAGCTTAATGGCAAGTGAGGGCATGGACCTTGATTGTGAAGACTCTGGTCTGATGACTGACACCAATGACGTAATACACATCCTACAAGCGCGTATCTAAGAGCAATTAACGATTGACCTCTGGGAAGACGCAGAAGACTTGCTCAATGAATACTTT‑‑‑‑‑‑‑‑‑GAGGAAGT‑‑‑‑‑‑‑‑‑CGcacgacagaaagaaattgaccgcgctaaggcccgtaaagaacgtcacgaggggcgcttagaggcacgcagattcaaacgtcgcaaccgcaaggcacgtaaagcacacaaagctaagcgcgaaagaatgc                                                                                                                                                               <  1:329599‑M1/301‑130 (MQ=255)`  
                                                                                                                                   `cTTTAGCGTAATGGCAAGTGAGGGCATTGACCTTGAGTTCGAAGACTCTGGTCTGATGCCTGACACCAAGGACGTAATCCGCATCCTGCAAGCGCGTATCTATGAGCAATTAACGATTGACCTCTGGGAAGACGCAGAAGACTTGCTCAATGAATACTTG‑‑‑‑‑‑‑‑‑GAGGAAGT‑‑‑‑‑‑‑‑‑CGcacgacagaaagaaattgaccgcgctaaggcccgtaaagaacgtcacgaggggcgcttagaggcacgcagattcaaacgtcgcaaccgcaaggcacgtaaagaacacaaagctaagcgcgaaagaatgctt                                                                                                                                                             >  1:48759‑M1/1‑170 (MQ=255)`  
                                                                                                                                              `tGGCAAGTGAGGGCATTGACCTTGAGTTCGAAGACTCTGGTCTGATGCCTGACACCAAGGACGTAATCCGCATCCTGCAAGCGCGTATCTATGAGCAATTAACGATTGACCTCTGGGAAGACGCAGAAGACTTGCTCCATGAATACTTG‑‑‑‑‑‑‑‑‑GAGGAAGT‑‑‑‑‑‑‑‑‑CGcacgacagaaagaaattgaccgcgctaaggcccgtaaagaacgtcacgaggggcgcttagaggcacgcagattcaaacgtcgcaaccgcaaggcacgtaaagcacacaaagctaagcgcgaaagaatgcttgctgcgtggcg                                                                                                                                                  >  1:299329‑M1/1‑159 (MQ=255)`  
                                                                                                                                               `ggCAAGTGAGGGCATTGACCTTGAGTTCGAAGACTCGGGTCTGATGCCTGACACCAAGGACGTAATCCGCATTCTGAAAGCGCGTATCTATGAGCAATTAACGATTGACCTCTGGGAAGACGCAGAAGACTTGCTCAATGAATACTTGGAGGAAGGCGAGGAAGT‑‑‑‑‑‑‑‑‑CGcacgacagaaagaaattgaccgcgctaaggcccgtaaagaacgtcacgaggggcgcttagaggcacgcagattcaaacgtcgcaaccgcaaggcacgtaaagcacacaaagctaagcgcgaaagaatgcttgct                                                                                                                                                          <  1:221204‑M1/301‑135 (MQ=255)`  
                                                                                                                                                `gCAAGTGAAGGCATTGACCTTGAGTTCGAAGACTCTGGTCTGATGCCTGACACCAAGGACGTAATCCGAATCCTGCTAGCGCGTATCTATGAGCAATTAACGATTGAGCTCTGGGAAGACGCAGAAGACTTGCTCAATGAATACTTGGAGGAAGTCGAGGAAGT‑‑‑‑‑‑‑‑‑CGcacgacagaaagaaattgaccgcgctaaggcccgtaaagaacgtcacgaggggcgcttagaggcacgcagattcaaacgtcgcaaccgcaaggcacgtaaagcacacaaagctaagcgcgaaagaatgcttgctg                                                                                                                                                         <  1:469079‑M1/301‑136 (MQ=255)`  
                                                                                                                                                   `aGTGAGGGCATTGACCTTGAGTTCGAAGACTCTGGTCTGATGCCTGACACCAAGGACGTAATCCGCATCCTGCAAGCGCGTATCTATGAGCAATTAACGATTGACCTCTGGGAAGACGCAGAAGACTTGCTCAATGAATACTTG‑‑‑‑‑‑‑‑‑GAGGAAGT‑‑‑‑‑‑‑‑‑CGcacgacagaaagaaattgaccgcgctaaggcccgtaaagaacgtcacgaggggcgcttagaggcacgcagattcaaacgtcgcaaccgcaaggcacgtaaagcacacaaagctaagcgcgaaagaatgcttgctgcgtggcgatggg                                                                                                                                             >  1:75848‑M1/1‑154 (MQ=255)`  
                                                                                                                                                         `ggCATTGACCTTGAGTTCGAAGACTCTGGTCTGATGCCTGACACCAAGGACGTAATCCGCATCCTGCAAGCGCGTATCTATGAGCAATTAACGATTGACCTCTGGGAAGACGCAGAAGACTTGCTCAATGAATACTTG‑‑‑‑‑‑‑‑‑GAGGAAGT‑‑‑‑‑‑‑‑‑CGcacgacagaaagaaattgaccgcgctaaggcccgtaaagaacgtcacgaggggcgcttagaggcacgcagattcaaacgtcgcaaccgcaaggcacgtaaagcacacaaagctaagcgcgaaagaatgcttgctgcgtggcgatgggctgaac                                                                                                                                       >  1:228375‑M1/1‑148 (MQ=255)`  
                                                                                                                                                         `ccggagatgtgtataagagacagctctggtcggatgcctgacaccaaggaCGTAATCCGCATCCTGCAAGCGCGTATCTATGAGCAATTAACGATTGACCTCTGGGAAGACGCAGAAGACTTGCTCAATGAATACTTG‑‑‑‑‑‑‑‑‑GAGGAAGT‑‑‑‑‑‑‑‑‑CGcacgacagaaagaaattgaccgcgctaaggcccgtaaagaacgtcacgaggggcgcttagaggcacgcagattcaaacgtcgcaaccgcaaggcacgtaaagcacacaaagctaagcgcgaaagaatgcttgctgcgtggcgatgggctgaac                                                                                                                                       <  1:159260‑M1/278‑154 (MQ=255)`  
                                                                                                                                                           `cATTGACCTTGAGTTCGAAGACTCTGGTCTGATGCCTGACACCAAGGACGTAATCCGCATCCTGCAAGCGCGTATCTATGAGCAATTAACGATTGACCTCTGGGAAGACGCAGAAGACTTGCTCAATGAATACTTG‑‑‑‑‑‑‑‑‑GAGGAAGT‑‑‑‑‑‑‑‑‑CGcacgacagaaagaaattgaccgcgctaaggcccgtaaagaacgtcacgaggggcgcttagaggcacgcagattcaaacgtcgcaaccgcaaggcacgtaaagcacacaaagctaagcgcgaaagaatgcttgctgcgtggcgatgggctgaacgt                                                                                                                                     >  1:549315‑M1/1‑146 (MQ=255)`  
                                                                                                                                                             `atgAACTTGAGTTCAGAGAATCTGGTCTGAGGGCTGACACCAAGGACGTAATCCGCACCCTGAAAGCGCGTATCTAAGAGGAATTAACGATTGACCTCTGGGAAGACGCAGAAGAATTGCTCAATGAATACGTGGAGGAAGGCGAGGAAGT‑‑‑‑‑‑‑‑‑CGcacgacagaaagaaattgaccgcgctaaggcccgtaaagaacgtcacgaggggcgcttagaggcacgcagattcaaacgtcgcaaccgcaaggcacgtaaagcacacaaagctaagcgcgaaagaatgcttgctgcgtggcgatgggc                                                                                                                                            <  1:628228‑M1/300‑149 (MQ=255)`  
                                                                                                                                                                  `ggctcggagatgtgtataagagacaggcctgacaccaaggacgtaatccgcatCCTGCAAGCGCGTATCTATGAGCAATTAACGATTGACCTCTGGGAAGACGCAGAAGACTTGCTCAATGAATACTTGGAGGAAGTCGAGGAAGT‑‑‑‑‑‑‑‑‑CGcacgacagaaagaaattgaccgcgctaaggcccgtaaataacgtcacgaggggcgcttagaggcacgcagattcaaacgtcgcaaccgcaaggcacgtaaagcacacaaagctaagcgcgaaagaatgcttgctgcgtggcgatgggctgaac                                                                                                                                       <  1:170893‑M1/275‑154 (MQ=255)`  
                                                                                                                                                                          `cGATGACTCTGGTCTGATGCCTGACACCAAGGACGTAATCCGCATCCTGCAAGCGCGTATCTATGAGAAATTAACGATTGACCTCTGGGAAGACGCAGAAGACTTGCTCAATGAATACTTG‑‑‑‑‑‑‑‑‑GAGGAAGT‑‑‑‑‑‑‑‑‑CGcacgacagaaagaaattgaccgcgctaaggcccgtaaagagcgtcacgaggggcgcttagaggcacgcagattcaaacgtcgcaaccgcaaggcacgtaaagcacacaaagctaagcgcgaaagaatgcttgctgcgtggcgatgggctgaacgtcaagaacggcgtaac                                                                                                                      <  1:112280‑M1/301‑171 (MQ=255)`  
                                                                                                                                                                          `cGAAGACTCTGGTCTGATGCCTGACACCAAGGACGTAATCCGCATCCTGCAAGCGCGTATCTATGAGCAATTAACGATTGACCTCTGGGAAGACGCAGAAGACTTGCTCAATGAATACTTG‑‑‑‑‑‑‑‑‑GAGGAAGT‑‑‑‑‑‑‑‑‑CGcacgacagaaagaaattgaccgcgctaaggcccgtaaagaacgtcacgaggggcgcttagagtcacgcagattcaaacgtcgcaaccgcaaggcacgtaaagcacacaaagctaagcgcgaaagaatgcttgctgcgtggcgatgggctgaacgtcaagaccggcgtaac                                                                                                                      >  1:462236‑M1/1‑131 (MQ=255)`  
                                                                                                                                                                            `aatactcTGGTCTGATGCCGGACCCCAAGGACGTAACCCGCATCCTGCAAGCGCGTATCTATGAGCAATTAACGATTGACCTCTGGTAAGACGCAGAAGACTTGCTCAATGAATACTTG‑‑‑‑‑‑‑‑‑GAGGAAGT‑‑‑‑‑‑‑‑‑CGcacgacagaaagaaattgaccgcgctaaggcccgtaaagaacgtcacgaggggcgcttagaggcacgcagattcaaacgtcgcaaccgcaaggcacgtaaagcacacaaagctaagcgcgaaagaatgcttgctgcgtggcgatgggctgaacgtcaagaacggcgtaacca                                                                                                                    <  1:342254‑M1/298‑173 (MQ=255)`  
                                                                                                                                                                            `aaGACGCTGGTCTGATGCCGGAAACCAAGAAGGTAATCCGCATGCTGCAAGGGCGTATCTATGAGCAATTAACGATTGACCTCTGGGAAGACGCAGAAGACTTGCTCAATGAATACTTG‑‑‑‑‑‑‑‑‑GAGGAAGT‑‑‑‑‑‑‑‑‑CGcacgacagaaagaaattgaccgcgctaaggcccgtaaagaacgtcacgaggggcgcttagaggcacgcagattcaaacgtcgcaaccgcaaggcacgtaaagcacacaaagctaagcgcgaaagaatgcttgctgcgtggcgatgggctgaacgtcaagaacggcgtaacca                                                                                                                    <  1:385568‑M1/301‑173 (MQ=255)`  
                                                                                                                                                                              `gACTCTGGTCTGATGCCTGACACCAAGGACGTAATCCGCATCCTGCAAGCGCGTATCTATGAGCAATTAACGATTGACCTCTGGGAAGACGCAGAAGACTTGCTCAATGAATACTTG‑‑‑‑‑‑‑‑‑GAGGAAGT‑‑‑‑‑‑‑‑‑CGcacgacagaaagaaattgaccgcgctaaggcccgtaaagaacgtcacgaggggcgcttagaggcacgcagattcaaacgtcgcaaccgcaaggcacgtaaagcacacaaagctaagcgcgaaagaatgcttgctgcgtggcgatgggctgaacgtcaagaacggcgtaaccatg                                                                                                                  >  1:235747‑M1/1‑127 (MQ=255)`  
                                                                                                                                                                                    `ggTCTGATGCCTGACACCAAGGACGTAATCCGCATCCTGCAAGCGCGTATCTATGAGCAATTAACGATTGACCTCTGGGAGGACGCAGAAGACTTGCTCAATGAATACTTG‑‑‑‑‑‑‑‑‑GAGGAAGT‑‑‑‑‑‑‑‑‑CGcacgacagaaagaaattgaccgcgctaaggcccgtaaagaacgtcacgaggggcgcttagaggcacgcagattcaaacgtcgcaaccgcaaggcacgtaaagcacacaaagctaagcgcgaaagaatgcttgctgcgtggcgatgggctgaacgtcaagaacggcgtaaccatgaggtct                                                                                                            >  1:81570‑M1/1‑121 (MQ=255)`  
                                                                                                                                                                                    `ggTCTGATGCCTGACACCAAGGACGTAATCCGCATCCTGCAAGCGCGTATCTATGAGCAATTAACGATTGACCTCTGGGAAGACGCAGAAGACTTGCTCAATGAATACTTG‑‑‑‑‑‑‑‑‑GAGGAAGT‑‑‑‑‑‑‑‑‑CGcacgacagaaagaaatttaccgcgctaaggcccgtaaagaacgtcacgatgggcgcttagaggcacgcagattcaaacgtcgcaaccgcacggcacgtaaagcacacaaagctaagcgcgaaagaatgcttgctgcgtggcgatgggctgaacgtcaagaacggcgtaaccatgaggtat                                                                                                            >  1:252108‑M1/1‑121 (MQ=255)`  
                                                                                                                                                                                    `ggTCTGATGCCTGACACCAAGGACGTAATCCGCATCCTGCAAGCGCGTATCTATGAGCAATTAACGATTGACCTCTGGGAAGACGCAGAAGACTTGCTCAATGAATACTTG‑‑‑‑‑‑‑‑‑GAGGAAGT‑‑‑‑‑‑‑‑‑CGcacgacagaaagaaattgaccgcgctaaggcccgtaaagaacgtcacgaggggcgcttagaggcacgcagattcaaacgtcgcaaccgcaaggcacgtaaagcacacaaagctaagcgcgaaagaatgcttgctgcgtggcgatgggctgaacctgtctcttatacacatctccgagccc                                                                                                            >  1:369676‑M1/1‑121 (MQ=255)`  
                                                                                                                                                                                      `tCTGATGCCTGACACCAAGGACGTAATCCGCATCCTGCAAGCGCGTATCTATGAGCAATTAACGATTGACCTCTGGGAAGACGCAGAAGACTTGCTCAATGAATACTTG‑‑‑‑‑‑‑‑‑GAGGAAGT‑‑‑‑‑‑‑‑‑CGcacgacagaaagaaattgaccgcgctaaggcccgtaaagaacgtcacgaggtgcgcttagaggcacgcagattcaaacgtcgcaaccgaaaggcacgtaaagcacaccaagctaagcgcgaaagaatgcttgctgcgtggcgatgggctgaacggcaagaacggcgtaaccatgagttagct                                                                                                          >  1:561988‑M1/1‑119 (MQ=255)`  
                                                                                                                                                                                            `gCCTGACACCAAGGACGTAATCCGCATCCTGCAAGCGCGTATCTATGAGCAATTAACGATTGACCTCTGGGAAGACGCAGAAGACTTGCTCAATGAATACTTG‑‑‑‑‑‑‑‑‑GAGGAAGT‑‑‑‑‑‑‑‑‑CGcgcgacagaaagaaattgaccgcgctaaggcccgtaaagaacgtcacgaggggcgcttagaggcacgcagattcaaacgtcgcaaccgcaaggcacgtaaagcacacaaagctaagcgcgaaagaatgcttgctgcgtggcgatgggctgaacgtcaagaacggcgtaaccatgaggtagctgtagat                                                                                                    >  1:294133‑M1/1‑113 (MQ=255)`  
                                                                                                                                                                                            `gCCTGACACCAAGGACGTAATCCGCATCCTGCAAGCGCGTATCTATGAGCAATTAACGATTGACCTCTGGGAAGACGCAGAAGACTTGCTCAATGAATACTTG‑‑‑‑‑‑‑‑‑GAGGAAGT‑‑‑‑‑‑‑‑‑CGcacgacagaaagaaattgaccgcgctaaggcccgtaaagaacgtcacgaggggcgcttagaggcacgcagattcaaacgtcgcaaccgcaaggcacgtaaagcacacaaagctaagcgcgaaagaatgcttgctgcgtggcgatgggctgaacgtcaagaacggcgtaaccatgaggtagctgtagat                                                                                                    >  1:555770‑M1/1‑113 (MQ=255)`  
                                                                                                                                                                                             `ccTGACACCAAGGACGTAAGCCGCATCCTGCAAGCGCGTATCTATGAGCAATTAATGATTGACCTCTGGGAAGACGCAGAAGACTTGCTCAATGAATACTTG‑‑‑‑‑‑‑‑‑GAGGAAGT‑‑‑‑‑‑‑‑‑CGcacgacagaaagaaattgaccgcgctaaggcccgtaaagaacgtcacgaggggcgcttagaggcacgcagattcaaacgtcgcaaccgcaaggcacgtaaagcacacaaagctaagcgcgaaagaatgcttgctgcgtggcgatgggctgaacgtcaagaacggcgtaaccatgaggtagctgtagatg                                                                                                   <  1:31695‑M1/301‑190 (MQ=255)`  
                                                                                                                                                                                                 `acacCAAGGACGTAATCCGCATCCTGCAAGCGCGTATCTATGAGCAATTAACGATTGACCTCTTGGAAGACGCAGAAGACTTGCTCAATGAATACTTG‑‑‑‑‑‑‑‑‑GAGGAAGT‑‑‑‑‑‑‑‑‑CGcacgacagaaagaaattgaccgcgctaaggcccgtaaagaacgtcacgaggggcgcttagaggcacgcagattcaaacgtcgcaaccgcaaggcacgtaaagcacacaaagctaagcgcgaaagaatgcttgctgcgtggcgatgggctgaacgtcaagaacggcgtaaccatgctgtctcttatacacatct                                                                                               >  1:24698‑M1/1‑108 (MQ=255)`  
                                                                                                                                                                                                 `acacCAAGGACGTAATCCGCATCCTGCAAGCGCGTATCTATGAGCAATTAACGATTGACCTCTGGGAAGACGCAGAAGACTTGCTCAATGAATACTTG‑‑‑‑‑‑‑‑‑GCGGAAGT‑‑‑‑‑‑‑‑‑CGcacgacagaaagaaattgaccgcgctaaggcccgtaaagaacgtcacgaggggcgcttagaggcacgcagattcaaacgtcgcaaccgcaaggcacgtaaagcacacaaagctaagcgcgaaagaatgcttgcggcgtggcgatgggctgaaagtcaagaacggcgtaaccataaggtagctgtagaggtact                                                                                               >  1:86768‑M1/1‑108 (MQ=255)`  
                                                                                                                                                                                                     `cagggacgTAATCCGAATCCTGAAAGCGCGTATCTATGAGCAATTAACGATGGACCTCTGGGAAGACGCAGAAGACTTGCTAAATGAATACTTG‑‑‑‑‑‑‑‑‑GAGGAAGTCGAGGAAGGCGcacgacagaaagaaattgaccgcgctaaggcccgtaaagaacgtcacgaggggcgcttagaggcacgcagattcaaacgtcgcaaccgcaaggcacgtaaagcacacaaagctaagcgcgaaagaatgcttgctgcgtggcgatgggctgaacgtcaagaacggcgtaaccatgaggtagctgtagat                                                                                                    <  1:373925‑M1/298‑189 (MQ=255)`  
                                                                                                                                                                                                     `cAAGGACGTAATCCGCATCCTGCAAGCGCGTATCTATGAGCAATTAACGATTGACCTCTGTGAAGACGCAGAAGACTTGCTCAATGAATACTTG‑‑‑‑‑‑‑‑‑GAGGAAGT‑‑‑‑‑‑‑‑‑CGcacgacagaaagaaattgaccgcgctgaggcccgtaaagaacgtcacgaggggcgcttagaggcacgcagattcaaacgtcgcaaccgcaaggcacgtaaagcacacaaagctaagcgcgaaagaatgcttgctgcgtggcgatgggctgaacgtcaagaacggcgtaaccatgaggtagctgtagatgtactagga                                                                                           <  1:577084‑M1/301‑198 (MQ=255)`  
                                                                                                                                                                                                     `cAAGGACGGAATCCGCATTCTGCAAGCGCGTATCTATGAGCAATTAACGATTGACCTCTGGGAAGACGCAGAAGACTTGCTCAATGAATACTTG‑‑‑‑‑‑‑‑‑GAGGAAGT‑‑‑‑‑‑‑‑‑CGcacgacagaaagaaattgaccgcgctaaggcccgtaaagaacgtcacgaggggcgcttagaggcacgcagattcaaacgtcgcaaccgtaaggcacgtaaagcacacaaagctaagcgcgaaagaatgcttgctgcgtggcgatgggctgaacgtcaagaacggcgtaaccatgaggtagctgtagatgtactagga                                                                                           <  1:643187‑M1/301‑198 (MQ=255)`  
                                                                                                                                                                                                     `aaagGACGTAATCCGCATCCTGCAAGCGCGTATCTATGAGCAATTAACGATTGACCTCTGGGAAGACGCAGAAGACTAGCTCAATGAATACTTG‑‑‑‑‑‑‑‑‑GAGGAAGT‑‑‑‑‑‑‑‑‑CGcacgacagaaagaaattgaccgcgctaaggcccgtaaagaacgtcacgaggggcgcttagaggcacgcagattcaaacgtcgcaaccgcaaggcacgtaaagcacacaaagctaagcgcgaaagaatgcttgctgcgtggcgatgggctgaacgtcaagaacggcgtaaccatgaggtagctgtagatgtactagga                                                                                           <  1:611087‑M1/300‑198 (MQ=255)`  
                                                                                                                                                                                                              `aaTCCGCATCCTGCAATCGCGTATCTATGAGCAATTAACGATTGACCTCTGGGAAGACGCAGAAGACTTGCTCAATGAATACTTG‑‑‑‑‑‑‑‑‑GAGGAAGT‑‑‑‑‑‑‑‑‑CGcacgacagaaagaaattgaccgcgctaaggcccgtaaagaacgtcacgaggggcgcttagaggcacgcagattcaaacgtcgcaaccgcaaggcacgtaaagcacacaaagctaagcgcgaaagaatgcttgctgcgtggcgatgggctgaacgtcaagaacggcgtaaccatgaggtagctgtagatgtacctgtctcttataca                                                                                  >  1:638384‑M1/1‑95 (MQ=255)`  
                                                                                                                                                                                                                `tCCGAATCCTGCAAGCGCGTATCTTTGAGCAATTAACGATTGACCTCTGGGAAGACGCAGAAAACTTGCTAAATAAATACTTGGAGGAAGTCGAGGAAGT‑‑‑‑‑‑‑‑‑CGcacgacagaaataaattgaccgcgctaaggcccgtaaagaacgtcacgatgggcgcgtagaggcacgcagattcaaacgtcgaaaccgcaaggcacgtaaagcacacaaagctaagcgcgaaagagtgcttgctgcgtggcgatgggctgaacgtcaagaacggcgtaaccatgaggtagctgtagatgtactaggaag                                                                                         <  1:669032‑M1/301‑200 (MQ=255)`  
                                                                                                                                                                                                                   `gcactctgcaaGCGCGTATCTATGAGCAATTAACGATTGACGTCTGGGAAGACGCAGAAGACTTGCGAAATGAATACTTG‑‑‑‑‑‑‑‑‑GAGGAAGT‑‑‑‑‑‑‑‑‑CGcacgacaaaaagaaattgaccgcgctaaggccagtaaagaccgtcacgaggggcgcttagaggcacgcagattcaaacgtcgcaaccgcaaggcacgtaaagcacacaaagctaagcgcgaaagaatgcttgctgcgtggcgatgggctgaacgtcaagaacggcgtaaccatgaggtagctgtagatgtactaggaagaaccaataacgc                                                                             <  1:444262‑M1/296‑212 (MQ=255)`  
                                                                                                                                                                                                                        `cTGCAAGCGCGTATCTATGAGCAATTAACGATTGACCTCTGGGAAGACGCAGAAGACTTGCTCAATGAATACTTG‑‑‑‑‑‑‑‑‑GAGGAAGT‑‑‑‑‑‑‑‑‑CGcacgacagaaagaaattgaccgcgctaaggcccgtaaagaacgtcacgaggggcgcttagaggcacgcagattcaaacgtcgcaaccgcaaggcacgtaaagcacacaaagctaagcgcgaaagaatgcttgctgcgtggcgatgggctgcacgtcaagaacggcgtaaccatgcggtagctgtagatgtactaggaagaaccaataacgctatgc                                                                        >  1:257616‑M1/1‑85 (MQ=255)`  
                                                                                                                                                                                                                                `gcgTATCTATTAGCAATTAACGATTGACCTCTGGGAAGACGCAGAAGACGTGCTCAATGAATACTTG‑‑‑‑‑‑‑‑‑GAGGAAGT‑‑‑‑‑‑‑‑‑CGcacgacagaaagaaatggaccgcgctaaggcccgtaaagaacgtcacgaggggcacttagaggcacgcagattcaaacgtcgcaaccgcaaggcacgtaaagcacacaaagctaagcgcgaaagaatgcttgctgcgtggcgatgggctgaacgtcaagaacggcgtaaccatgaggtagctgtagatgtactaggaagaaccaataacgctatgctctgggtc                                                                <  1:275327‑M1/301‑225 (MQ=255)`  
                                                                                                                                                                                                                                    `aTCTATGAGCAATTAACGATTGACCTCTGGGAAGACGCAGAAGACTTGCTCAATGAATACTTG‑‑‑‑‑‑‑‑‑GAGGAAGT‑‑‑‑‑‑‑‑‑CGcacgacagaaagaaattgaccgcgctaaggcccgtaaagaacgtcacgaggggcgcttagaggcacgcagattcaaacgtcgcaaccgcaaggcacgtaaagcacacaaagctaagcgcgaaagaatgcttgctgcgtggcgatgggctgaacgtcaagaacggcgtaaccatgaggtagctgtagatgtactaggaagaaccaataacgctatgctctgggtcaaca                                                            >  1:584151‑M1/1‑73 (MQ=255)`  
                                                                                                                                                                                                                                     `tCTATGAGCAATTAACGATTGACCTCTGGGAAGACGCAGAAGACTTGCTCAATGAATACTTG‑‑‑‑‑‑‑‑‑GAGGAAGT‑‑‑‑‑‑‑‑‑CGcacgacagaaagaaattgaccgcgctaaggcccgtaaagaacgtcacgaggggcgcttagaggcacgcagattcaaacgtcgcaaccgcaaggcacgtaaagcacacaaagctaagcgcgaaagaatgcttgctgcgtggcgatgggctgaacgtcaagaacggcgtaaccatgaggtagctgtagatgtactaggaagaaccaataacgctatgctctgggtcaacat                                                           <  1:423704‑M1/301‑230 (MQ=255)`  
                                                                                                                                                                                                                                     `tCTATGAGCAATTAACGATTGACCTCTGGGAAGACGCAGAAGACTTGCTCAATGAATACTTG‑‑‑‑‑‑‑‑‑GAGGAAGT‑‑‑‑‑‑‑‑‑CGcacgacagaaagaaattgaccgcgctaaggcccgtaaagaacgtcacgaggggcgcttagaggcacgcagattcaaacgtcgcaaccgcaaggcacgtaaagcacacaaagctaagcgcgaaagaatgcttgctgcgtggcgatgggctgaacgtcaagaacggcgtaaacatgaggtagctgtagatgtactaggaagaaccaataacgctatgctctgggtcaacat                                                           >  1:400398‑M1/1‑72 (MQ=255)`  
                                                                                                                                                                                                                                           `agaaattaACGATGGACCTCTGGGAAGACGCAGAAGACTTTCTCAATGAATACTTG‑‑‑‑‑‑‑‑‑GAGCAAGT‑‑‑‑‑‑‑‑‑CGgacgacagaaagaaatggaccgcgctaaggcccgtaaagaacgtcacgaggggcgcttagaggcacgcagattcaaacgtcgcaaccgcaaggcacgtaaagcacacaaagctaatcgcgaaagaatgcttgctgcgtggcgatgggctgaacgtcaagaacggcgtaaccatgaggtagctgtagatgtactaggaagaaccaataacgctatgctctgggtcaacatgttctc                                                     <  1:336267‑M1/298‑236 (MQ=255)`  
                                                                                                                                                                                                                                           `aGCAATTAACGATTGACCTCTGGGAAGACGCAGAAGACTTGCTCAATGAATACTTG‑‑‑‑‑‑‑‑‑GAGGAAGT‑‑‑‑‑‑‑‑‑CGcacgacagaaagaaattgaccgcgctaaggcccgtaaagaacgtcacgaggggcgcttagaggcacgcagattcaaacgtcgcaaccgcaaggcacgtaaagcacacaaagctaagcgcgaaagaatgcttgctgcgtggcgatgggctgaacgtcaagaacggcgtaaccatgaggtagctgtagatgtactaggaagaaccaataacgctatgctctgggtcaacatgttctc                                                     >  1:499225‑M1/1‑66 (MQ=255)`  
                                                                                                                                                                                                                                            `gCAATTACAGATTGACCTATGGGAAGACGCAGAAGACTTGCTCAATGAATACTTG‑‑‑‑‑‑‑‑‑GAGGAAGT‑‑‑‑‑‑‑‑‑CGaggaagtcgaacgacagtaagaaattgcccgcgctaaggcccgtaaagaacgtcaacaggggcgcttagaggcacgcagattcaaacgtcgcaaccgcaaggcacgtaaagcacacaaagctaagcgcgaaagaatgctttctgcgtggcgatgggctgaacgtcaagaacggcgtaaccattaggtagctgtagatgtactaggaagaaccaataacgctatgctctgggtcaac                                                    <  1:359756‑M1/301‑237 (MQ=255)`  
                                                                                                                                                                                                                                              `aagtaacGATTGACCGCTGGGAAGACACAGAAGACTAGGTAAATGAATAATTGGAGGAAGTCGAGGAAGT‑‑‑‑‑‑‑‑‑CGcacgacagaaagaaattgaccgcgctaaggcccgtaaagaacgtaacgaggggcgcttagaggcacgcagattcaaacgtcgcaaccgcaaggcacgtaaagcacacaaagctaagcgcgaaagaatgcttgctgcgtggcgatgggctgaacgtcaagaacggcgtaaccatgaggtagctgtagatgtactaggaagaaccaataacgctatgctctgggtcaacat                                                           <  1:633273‑M1/298‑230 (MQ=255)`  
                                                                                                                                                                                                                                               `aTTAACGATTGACCTCTGGGAAGACGCAGAAGACTTGCTCAATGAATACTTGGAGGAAGTCGAGGAAGT‑‑‑‑‑‑‑‑‑CGcacgacagaaagaaattgaccgcgctaaggcccgtaaagaacgtcacgaggggcgcttagaggcacgcagattcaaacgtcgcaaccgcaaggcacgtaaagcacacaaagctaagcgcgaaagaatgcttgctgcgtggcgatgggctgaacgtcaagaacggcgtaaccatgaggtagctgtagatgtactaggaagaaccaataacgctatgctctgggtcaacatg                                                          >  1:364423‑M1/1‑71 (MQ=255)`  
                                                                                                                                                                                                                                               `aTTAACGATTGACCTCTGGGAAGACGCAGAAGACTTGCTCAATGAATACTTG‑‑‑‑‑‑‑‑‑GAGGAAGT‑‑‑‑‑‑‑‑‑CGcacgacagaaagaaattgaccgcgctaaggcccgtcaagaacgtcacgaggggcgcttagaggcacgcagattcactcgtcgcaaccccaaggcacgtaaagcacacaaagctaagcgcgaaagaatgcttgctgcgtggcgatgggctgaacgtcaagaacggcgtaagcatgaggtagctgtagatgtactaggaagaaccaataacgctatgctctggacccacattttctctggg                                                 >  1:664442‑M1/1‑62 (MQ=255)`  
                                                                                                                                                                                                                                               `aTTAACGATTGACCTCTGGGAAGACGCAGAAGACTTGCTCAATGAATACTTG‑‑‑‑‑‑‑‑‑GAGGAAGT‑‑‑‑‑‑‑‑‑CGcacgacagaaagaaattgaccgcgctaaggcccgtaaagaacgtcacgaggggcgcttagaggcacgcagattcagacgtcgcaaccgcaaggcacgtaaagcacacaaagctaagcgcgaaagaatgcttgctgcgtggcgatgggctgaacgtcaagaacggcgtaaccatgaggtagctgtagatgtactaggaagaaccaataacgcctggctcttatacacatctccgagccca                                                 >  1:281119‑M1/1‑62 (MQ=255)`  
                                                                                                                                                                                                                                                   `aCGATTGACCTCTGGGAAGACGCAGAAGACTTGCTCAATGAATACTTG‑‑‑‑‑‑‑‑‑GAGGAAGT‑‑‑‑‑‑‑‑‑CGcacgacagaaagaaattgaccgcgctaaggcccgtaaagaacgtcacgaggggctcttagaggcacgcagattcaaacgtcgcaaccgcaaggcacgtaaagcacacaaagctaagcgcgaaagaatgcttgctgcgtggcgatgggctgaacatcaggaacggcgtaaccatgaggtagctgtagatgtactaggaagaaccaataacgctatgctctgggtcaacatgttctctggggact                                             <  1:435599‑M1/301‑244 (MQ=255)`  
                                                                                                                                                                                                                                                    `cGATTGCCCTCGGGGAAGACGCAGAAGACTTGCTCAATGAATACTTGGAGGAAGTCGAGGAAGT‑‑‑‑‑‑‑‑‑CGcacgaaagaaagaaattgaccgcgctaaggcccgtaaagaacgtcacgaggggcgcttagaggcacgcagattcaaacgtcgcaaccgcaaggcacgtaaagcacacaaagctaagcgcgaaagaatgcttgctgcgtggcgatgggctgaacgtcaagaacggcgtaaccatgaggtagctgtagatgtactaggaagaaccaataacgctatgatctgggtcaacatgttctc                                                     <  1:165113‑M1/301‑236 (MQ=255)`  
                                                                                                                                                                                                                                                    `cGATTGACCGCTGGGAAGACGCAGAAGACTTGCTCAATGAATACTTGGAGGAAGTCGAGGAAGT‑‑‑‑‑‑‑‑‑CGcacgacagaaagaaattgaccgcgctaaggcccgtaaagaacgtcacgaggggcgcttagaggcacgcagattcaaacgtcgcaaccgcaaggcacgtaaagcacacaaagctaagcgcgaaagaatgcttgctgcgtggcgatgggctgaacgtcaagaacggcgtaaccatgaggtagctgtagatgtactaggaagaaccaataacgctatgctctgggtcaacatgttctc                                                     <  1:658772‑M1/301‑236 (MQ=255)`  
                                                                                                                                                                                                                                                         `gACCTCTGGGAAGACGCAGAAGACTTGCTCAATGAATACTTG‑‑‑‑‑‑‑‑‑GAGGAAGT‑‑‑‑‑‑‑‑‑CGcacgacagaaagaaattgaccgcgctaaggcccgtaaagaacgtcacgaggggcgcttagaggcacgcagattcaaacgtcgcaaccgcaaggcacgtaaagcacacaaagctaagcgcgaaagaatgcttgctgcgtggcgatgggctgaacgtcaagaacggcgtaaccatgaggtagctgtagatgtactaggaagaaccaataacgctatgctctgggtcaacatgttctctggggactttatgg                                       <  1:596765‑M1/301‑250 (MQ=255)`  
                                                                                                                                                                                                                                                              `ctGGGAAGACGCAGAAGACTTGCTCAATGAATACTTGGAGGAAGTCGAGGAAGT‑‑‑‑‑‑‑‑‑CGcacgacagaaagaaatttaccgcgctacggcccgtaaagaacgtcacgacgggcgcttagagtcactcatattccaacgtccccaccgcaagtcacgtaaagcacacaacgctaagcgcgaaagaatgcttgctgcgtggcgatgggctgaacgtcaaaaacggcgtaaccatgaggtagctgtagatgtactcggaagaaccaataacgctatgctctgggtcaacatgttctctggggccttt                                           >  1:41406‑M1/1‑56 (MQ=255)`  
                                                                                                                                                                                                                                                                `gggAAGACGCAGAAGACTTGCTCAATGAATACTTG‑‑‑‑‑‑‑‑‑GAGGAAGT‑‑‑‑‑‑‑‑‑CGcacgacagaaagaaattgaccgcgctaaggcccgtaaagaacgtcacgagtggcgcttagaggtacgcagattcaaacgtcgcaaccgcaaggcacgtaaagcacacaaagctaagcgcgcaagaatgcttgctgcgtggcgatgggctgaacgtcaagaacggcgtaaccatgaggtagctgtagatgtactaggaagaaccaataacgctatgctctgggtcaacatgttcgctggggactttaaggcgcttga                                >  1:125806‑M1/1‑45 (MQ=255)`  
                                                                                                                                                                                                                                                                  `gAAGACGCAGAAGACTTGCTCAATGAATACTTG‑‑‑‑‑‑‑‑‑GAGGAAGT‑‑‑‑‑‑‑‑‑CGcacgaaagaaagaaattgaccgcgctaaggcccgtaaagaacgtcacgaggggcgcttagaggcacgcagattcaaacgtcgcaaccgcaaggcacgtaaagcacacaaagctaagcgcgaaagaatgcttgctgcgtggcgatgggctgaacgtcaagaacggcgtaaccatgaggtagctgtagatgtactaggaagaaccaataacgctatgctctgggtcaacatgttctctggggactttaaggcgcttgagg                              <  1:94019‑M1/301‑259 (MQ=255)`  
                                                                                                                                                                                                                                                                        `gCAGAAGACTTGCTCAATGAATACTTG‑‑‑‑‑‑‑‑‑GAGGAAGT‑‑‑‑‑‑‑‑‑CGcacgacagaaagaaattgaccgcgctaaggcccgtaaagaacgtcacgaggggcgcttagaggcacgcagattcaaacgtcgcaaccgcaaggcacgtaaagcacacaaagctaagcgcgaaagaatgcttgctgcgtggcgatgggctgaacgtcaagaacggcgtaaccatgaggtagctgtagatgtactaggaagaaccaataacgccatgctctgggtcaacatgttctctggggactttaaggcgcttgaggaactgt                        >  1:15371‑M1/1‑37 (MQ=255)`  
                                                                                                                                                                                                                                                                                   `gCTCAATGAATACTTG‑‑‑‑‑‑‑‑‑GAGGAAGT‑‑‑‑‑‑‑‑‑CGcacgacagaaagaaattgaccgcgctaaggcccgtaaagaacgtcacgaggggcgcttagaggcacgcagattcaaacgtcgcaaccgcaaggcacgtaaagcacacaaagctaagcgcgaaagaatgcttgctgcgtggcgatgggctgaacgtcaagaacggcgtaaccatgaggtagctgtagatgtactaggaagaaccaataacgctatgctctgggtcaacatgttctctggggactttaaggcgcttgaggaacgaatcgcgctgcac             <  1:536080‑M1/301‑276 (MQ=255)`  
                                                                                                                                                                                                                                                                                       `aaTGAATACTTG‑‑‑‑‑‑‑‑‑GAGGAAGT‑‑‑‑‑‑‑‑‑CGcacgacagaaagaaattgaccgcgctaaggcccgtaaagaacgtcacgaggggcgcttagaggcacgcagattcaaacgtcgcaaccgcaaggcacgtaaagcacacaaagctaagcgcgaaagaatgcttgctgcgtggcgatgggctgaacgtcaagaacggcgtaaccatgaggtagctgtagatgtactaggaagaaccaataacgctatgctctgggtcaacatgttctctggggcctttaaggcgcttgaggaacgaatcgcgctgcactggc         >  1:118815‑M1/1‑22 (MQ=255)`  
                                                                                                                                                                                                                                                                                       `aaTGAATACTTG‑‑‑‑‑‑‑‑‑GAGGAAGT‑‑‑‑‑‑‑‑‑CGcacgacagaaagaaattgaccgcgctaaggcccgtaaagaacgtcacgaggggcgcttagaggcacgcagattcaaacgtcgcaaccgcaaggcacgtaaagcacacaaagctaaacgcgaaagaatgcttgctgcgtggcgatgggctgaacgtcaagaacggcgtaaccatgaggtagctgtagatgtactaggaagaaccaataacgctatgctctgggtcaacatgttctctggggactttaaggcgcttgaggaacgaatcgcgctgcactggc         >  1:288934‑M1/1‑22 (MQ=255)`  
                                                                                                                                                                                                                                                                                        `aTGAATACTTG‑‑‑‑‑‑‑‑‑GAGGAAGT‑‑‑‑‑‑‑‑‑CGcacgacagaaagaaattgaccgcgctaaggcccgtaaagaacgtcacgaggggcgcttagaggcacgcagattcaaacgtcgcaaccgcaaggcacgtaaagcacacaaagctaagcgcgaaagaatgcttgctgcgtggcgatgggctgaacgtcaagaccggcgtaaccatgaggtagctgtagatgtactaggaagaaccaataacgctatgctctgggtcaacatgttctctggggactttaaggcgcttgaggaacgaatcgcgctgcactggcg        >  1:204862‑M1/1‑21 (MQ=255)`  
                                                                                                                                                                                                                                                                                        `aTGAATACTTG‑‑‑‑‑‑‑‑‑GAGGAAGT‑‑‑‑‑‑‑‑‑CGaacgacagaaagaaattgaccgcgctaaggcccgtaaagaacgtcacgaggggcgcttagaggcacgcagattcaaacgtcgcaaccgcaagtcacgtaaagcacacaaagctaagcgcgaaagaatgcttgctgcgtggcgatgggctgaacgtcaagaacggcgtaaccatgaggtagctgtagatgtactaggaagaaccaataacgctatgctctgggtcaacatgttctctggggactttaaggcgcttgaggaacgaatcgcgctgcactggcg        <  1:647786‑M1/301‑281 (MQ=255)`  
                                                                                                                                                                                                                                                                                             `tACTTG‑‑‑‑‑‑‑‑‑GAGGAAGT‑‑‑‑‑‑‑‑‑CGcacgacagaaagaaattgaccgctctaaggcccgtaaagaacgtcacgaggggcgcttagaggcacgcagattcaaacgtcgcaaccgcaaggcacgtaaagcacacaaagctaagcgcgaaagaatgcttgctgcgtggcgatgggctgaacgtcaagaacggcgtaaacatgaggtagctgtagatgtactaggaagaaccaataacgcaatgctctgggtcaacatgttctctggggactttaaggcgcttggggaacgaatcgcgctgaactggcgtaagg   >  1:517264‑M1/1‑16 (MQ=255)`  
                                                                                                                                                                                                                                                                                              `aCTTG‑‑‑‑‑‑‑‑‑GAGGAAGT‑‑‑‑‑‑‑‑‑CGcacgacagaaagaaattgaccgcgctaaggcccgtaaagaacgtcacgaggggcgcttagaggcacgcagattcaaacgtcacaaccgcaaggcacgtaaagcacacaaagctaagcgcggaagaatgcttgctgcgtggcgatgggctgaacgtcaagaacggcgtaaccatgaggtagctgtagatgtactaggaagaaccaataacgctatgctctgggtcaacatgttctctggggactttaaggcgcttgaggaacgaatctgtctcttatacacatctcc  >  1:510295‑M1/1‑15 (MQ=255)`  

                                                                                                                                                                                                                                                                                                                              `|`                                                                                                                                                                                                                                                                                                
`AACGTTTTCGACCACGCTTACGAAATGCTGAAAGAAAACATCCGTTATGATGACATCCGTGACACTGATGACCTGCACGATGCTATTCACATGGCTGCCGATAATGCAGTTCCGCACTACTACGCTGACATCTTTAGCGTAATGGCAAGTGAGGGCATTGACCTTGAGTTCGAAGACTCTGGTCTGATGCCTGACACCAAGGACGTAATCCGCATCCTGCAAGCGCGTATCTATGAGCAATTAACGATTGACCTCTGGGAAGACGCAGAAGACTTGCTCAATGAATACTTG‑‑‑‑‑‑‑‑‑GAGGAAGT‑‑‑‑‑‑‑‑‑CGAGGAGTACGAGGAGGATGAAGAGTAATGTCTACTACCAACGTGCAATACGGTCTGACCGCTCAAACTGTACTTTTCTATAGCGACATGGTGCGCTGTGGCTTTAACTGGTCACTCGCAATGGCACAGCTCAAAGAACTGTACGAAAACAACAAGGCAATAGCTTTAGAATCTGCTGAGTGATAGACTCAAGGTCGCTCCTAGCGAGTGGCCTTTATGATTATCACTTTACTTATGAGGGAGTAATGTATATGCTTACTATCGGTCTACTCACCGCTCTAGGTCTAG  >  NC_001604.1/952‑1538`

  

Alignment Legend

Aligned base mismatch/match (shaded by quality score): `ATCG/ATCG` `< 3 ≤` `ATCG/ATCG` `< 10 ≤` `ATCG/ATCG` `< 22 ≤` `ATCG/ATCG` `< 34 ≤` `ATCG/ATCG` `< 38 ≤` `ATCG/ATCG`

Unaligned base: `atcg`    Masked matching base: `atcg`    Alignment gap: `‑`    Deleted base: `‑`
